# The role of sex in the innate and adaptive immune environment of metastatic colorectal cancer

**DOI:** 10.1038/s41416-020-0913-8

**Published:** 2020-05-26

**Authors:** Anita L. Ray, Robert A. Nofchissey, Maaz A. Khan, Megan A. Reidy, Megan R. Lerner, Xiangyan Wu, Shaoxuan Guo, Spencer L. Hill, Nathaniel Weygant, Sarah F. Adams, Eliseo F. Castillo, William L. Berry, Michael B. Stout, Katherine T. Morris

**Affiliations:** 1 Department of Surgery, University of Oklahoma Health Sciences Center, Oklahoma City, OK USA; 2 Department of Medicine, University of Oklahoma Health Sciences Center, Oklahoma City, OK USA; 3 Fujian University of Traditional Chinese Medicine, Academy of Integrative Medicine, Fujian, China; 4 Department of Obstetrics & Gynecology, University of New Mexico, Albuquerque, NM USA; 5 Department of Internal Medicine, University of New Mexico, Albuquerque, NM USA; 6 Department of Nutritional Sciences, University of Oklahoma Health Sciences Center, Oklahoma City, OK USA

**Keywords:** Tumour immunology, Colorectal cancer, Tumour immunology, Chemokines

## Abstract

**Background:**

Women with colorectal cancer (CRC) have a significant survival advantage over men. Sex influences on the tumour microenvironment (TME) are not well characterised, despite the importance of immune response in CRC. We hypothesised that sex-divergent immune responses could contribute to survival.

**Methods:**

Using a murine model of metastatic CRC, we examined T cells, macrophages, and cytokines locally and systemically. TME and serum cytokines were measured by multiplex bead-based arrays, while FCA was used to identify cells and phenotypes. IHC provided spatial confirmation of T cell infiltration.

**Results:**

Females had increased survival and T cell infiltration. CD8, CD4 and Th2 populations correlated with longer survival. Males had increased serum levels of chemokines and inflammation-associated cytokines. Within the TME, males had lower cytokine levels than females, and a shallower cytokine gradient to the periphery. Female tumours had elevated IL-10+ macrophages, which correlated with survival.

**Conclusions:**

These data demonstrate survival-associated differences in the immune response of males and females to metastatic CRC. Females showed changes in cytokine production accompanied by increased immune cell populations, biased toward Th2-axis phenotypes. Key differences in the immune response to CRC correlated with survival in this model. These differences support a multi-faceted shift across the TME.

## Background

Colorectal cancer (CRC) is the third most frequent malignancy in men and women.^1^ Women are known to have a higher frequency of right sided tumours, are generally older at diagnosis, and most studies reveal longer overall and disease specific survival for women.^2–4^ The mechanisms behind the survival benefit seen in women are not clear. While previous investigators have examined the effects of sex steroids on the CRC cells themselves, little is known about whether men and women have a different immune response to CRC, although the immune response strongly influences outcomes. Recently, Immunoscore™, a measure of T cell infiltration, was shown to be the highest relative contributor to CRC recurrence risk of all currently known clinicopathologic parameters.^5^ Clearly, CRC is a malignancy where outcomes are not only determined by what is occurring in the cancer cells themselves, but also by the composition and function of the immune cells within the tumour microenvironment (TME). In addition, serum cytokine levels, such as IL-6, correlate with poor CRC outcomes, suggesting that a patient’s systemic inflammatory response to CRC may also be linked to their ability to survive the disease.^6^ Surprisingly, whether the composition of the TME or serum cytokine responses from patients with CRC differ based on sex is not known, despite the difference in survival noted in this disease.

While there is a key knowledge gap regarding the effects of sex on the immune response to CRC, there is evidence that sex affects immune response to other health-related conditions, including sepsis, vaccination responses and autoimmune disease. Specifically, the peritoneal cavity, a site of CRC metastasis, has been shown to have sex-specific differences in immune cell populations and responses in both healthy and pro-inflammatory states. For example, resident peritoneal macrophage and T cell populations are more abundant in healthy female mice than males.^7^ Furthermore, the introduction of bacteria in a model of peritoneal infection led to distinct responses, with female mice having decreased peritoneal inflammation, and yet increased macrophage and regulatory T cell responses. These findings correlated with improved bacterial clearance and reduced sepsis severity in females, supporting the hypothesis that the observed immune variation is likely to have clinically relevant impacts. Similar to CRC, women with sepsis survive longer than men.^8^ Sex-based differences in immune response are not confined to the peritoneal cavity. Men have been found to have an increased systemic Th1 response to several different vaccines, while women generally have increased Th2 responses.^9^ These reports are especially intriguing because cytokine secretion, immune infiltration, and immune polarisation have all been shown to affect outcomes in CRC. If sex changes these clinically relevant immune responses, it could be contributing to the survival differences seen between men and women with CRC. Perhaps even more importantly, understanding these differences could improve our ability to create a favourable immune environment to fight CRC.

The primary objective of this study was to test the hypothesis that there would be a sex-specific immune response to metastatic CRC in an immune competent orthotopic murine model of peritoneal carcinomatosis. The secondary objective was to determine if these differences correlated with survival. In this model of advanced CRC, we found significant changes in T cell infiltration and activity, systemic and TME cytokine responses, and phenotypic changes in tumour-associated macrophages when comparing males and females. Increased T cells and IL-10-producing macrophages in the TME correlated with survival as well. We also found that females had lower systemic levels of chemotactic and inflammatory cytokines, but higher levels produced within the TME. Our data suggest fundamentally different immune responses to CRC. These differences are closely tied to survival, supporting a potential cause for increased survival in women with CRC.

## Methods

### MC38 culture and tumour model

MC38 cells (gifted by Drs. Schulick and Barnett at the University of Colorado) were validated (DDC Medical, Fairfield OH, USA) and tested for mycoplasma (Idexx, Columbia MO, USA), then cultured at sub-confluency in RPMI-1640 with 10% FBS (Lonza, Basel, Switzerland), 1% penicillin-streptomycin (Lonza), and 1% l-glutamine (Thermofisher, Carlsbad CA, USA). This cell line originates from a chemically induced colon cancer in a female C57Bl/6 mouse.^10,11^ Husbandry details can be found in “Supplementary Methods”. For survival and flow cytometry analysis, 10^5^ cells from passage eight or less were injected intraperitoneally at noon on Day 0 into 6–8-week-old C57Bl/6J (B6J) mice obtained from The Jackson Laboratory (Bar Harbor ME, USA). Eight females and 10 males were used. Animals were euthanised when pre-determined objective criteria for morbidity were reached, or at day 23 post-injection. Animals were monitored 3×/week until symptoms developed, then daily. Morbidity was determined by evaluation of weight change compared to age and sex-matched controls, rapid weight loss/gain (10% change over 3 days), development of ascites, lethargy, and distress based on a grimace scale.^12^

For cytokine analysis of serum and tumours, whole blood and tumours were collected from animals injected with 10^6^ MC38 intraperitoneally as above. Tumour tissue normalised by weight (8 ± 1 mg) was incubated for 16 h in cell culture media. Collected media was analysed, along with serum, with a magnetic bead-based array (PCYTMAG-70K-PX32 Millipore Sigma, Burlington MA, USA) per manufacturer’s protocol. These animals had the same morbidity evaluation as the tumour model above; control mice were age- and sex-matched and injected with vehicle. In all, 18 tumour and 12 control mice were used (half male).

All procedures were approved by the Institutional Animal Care and Use Committee in accordance with existing national and university policy on humane care and use of laboratory animals.

### Tissue preparation

Spleens were mechanically dissociated in cell culture media. To obtain non-adherent peritoneal cells, peritoneal cavities were washed with DPBS as previously described.^13^ After sacrifice, the peritoneal cavity was injected with sterile DPBS, then massaged. The wash was collected and centrifuged at 300×*g* to collect cells. Red blood cells from the ascites were lysed using a buffer of ice-cold 155 mM NH_4_Cl, 12 mM NaHCO_3_, and 0.1 mM EDTA.

Tumours were dissociated using a gentleMACS Dissociator (Miltenyi Biotec, Bergisch Gladbach, Germany), then incubated at 5% CO_2_ and 37 °C in RPMI-1640 with 1% l-glutamine, 1% pen-strep, 10% FBS, and 20 μg/ml Liberase (Roche, Basel, Switzerland) for 40 m. Samples from each tissue were passed through 40 μM cell strainer (VWR, Radnor PA, USA).

### Immunohistochemistry

Four micron thick histological sections, embedded in paraffin and mounted on HistoBond^®^Plus slides (Statlab Medical Products, Lewisville TX, USA) were rehydrated and washed in TBS. Sections were processed using the ImmPRESS™ Goat Anti-Rat IgG (Mouse Adsorbed) HRP Polymer kit (Vector Labs, Burlingame CA, USA). Antigen retrieval (high pH tris antigen unmasking solution, Vector Labs) was accomplished via twenty minutes in a steamer followed by thirty minutes at room temperature. Sections were treated with a peroxidase blocking reagent (Bloxall, Vector Labs) to inhibit endogenous peroxidase activity, followed by 2.5% normal goat serum block. CD8a (4SM15) (eBioscience, San Diego CA, USA; 5 µg/ml) and CD4 (4SM95) (eBioscience; 5 µg/ml) antibodies were applied to each section. Following incubation overnight at 4 °C in a humidified chamber, sections were washed in TBS and the ImmPRESS Polymer reagent was applied according to the manufacturer’s directions.

Slides were incubated with NovaRed® (Vector Labs) for visualisation. Counterstaining was carried out with Methyl Green (Vector Labs). Appropriate positive and negative tissue controls were used.

Positive cells were counted per 40× field by an experimentally blinded researcher and independently verified by another. A minimum of 26 fields/sample and an average of 50 fields/sample were counted. Blood vessels, non-tumour tissue, or necrotic areas were excluded. Representative images were adjusted to correct colour and improve contrast using Photoshop Elements (Adobe, San Jose CA, USA). Originals available on request.

### Flow cytometric analysis (FCA)

Antibodies included anti-mouse IFNγ-R-PE (XMG1.2), IL-10-BV421 (JES5-16E3), IL-12p35-FITC (C15.6), IL-17A-Alexa488 (TC11-18H10), CD4-PerCP-Cy5.5 (RM4-5), CD8a-APC-Cy7 (53-6.7), CD11b-BV650 (M1/70), F4/80-Alexa647 (T45-2342) (BD Biosciences, San Jose CA, USA) and IL-4-Alexa647 (11B11, Biolegend, San Diego CA, USA). Isotype controls were sourced from the same manufacturer. Cells were gated for viability using Ghost Dye Violet 510 (Tonbo, San Diego CA, USA). Single colour controls were acquired using the above antibodies in combination with VersaComp Beads (Beckman Coulter, Pasadena CA, USA). Cells stained for intracellular cytokines were treated with GolgiPlug (BD Biosciences) per manufacturer protocol.

Staining was performed per standard protocol; cells were blocked with 10% rat serum and anti-CD16/CD32 Fc Block (BD Biosciences), then stained for 30 m at 4 °C in FCA buffer (DPBS, 0.5% bovine serum albumin, and 0.1% sodium azide). Cells were washed and fixed with BD Cytofix. For cytokines, cells were permeabilised in BD Cytoperm and stained as per above. Cells were run on a Stratedigm S1000EXi Flow Cytometer (San Jose CA, USA). Data was analysed using FlowJo v10.5.2 software (Treestar, Ashland OR, USA).

### Statistics

An *n* of 8–10 was used to detect effect sizes consistent with previous studies’ effect sizes. *p*-values were acquired using two-tailed Student *t*-tests if data were normally distributed or Mann–Whitney U test if not. Normality of data sets was determined by the Shapiro–Wilk normality test. Survival curves were analysed using log-rank (Mantel-Cox) testing. Using a two-sided log-rank test and alpha of 0.05, we had 85.0% power to detect a mortality ratio of 3.2, assuming 10 male and 8 female mice and 25% mortality in female mice, as seen at d20. *p*-values of ≤0.050 were considered significant. Data displayed shows the mean with standard deviation.

## Results

### Females survive longer and have increased CD8+ T cell infiltration in the TME

**Fig. 1:**
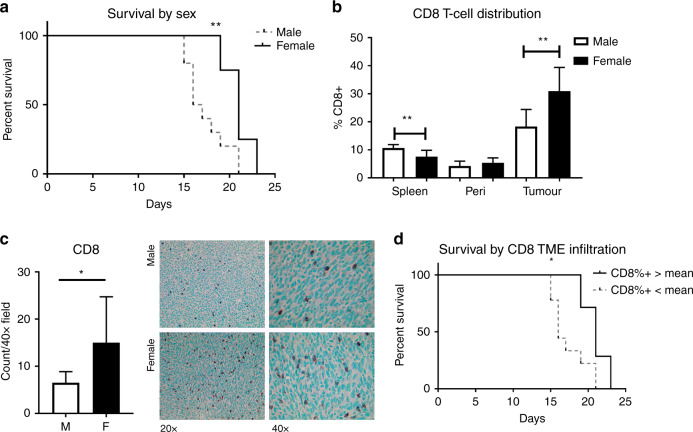
Females live longer than males in a metastatic model of CRC, and survival is correlated with increased CD8 T cells in the TME. 10^5^ MC38 cells were delivered intraperitoneally to syngeneic male and female mice. **a** Females had increased survival, and **b** a higher percentage of CD8+ T cells in the TIL population, as assessed by FCA. **c** IHC staining with anti-CD8 confirmed greater infiltration of CD8 + cells in females. **d** Mice were divided by CD8+ infiltration, as assessed by FCA, into high (above mean) and low (below mean) groups. High infiltration correlated with survival (*n* = 5–10). *p < 0.050, **p < 0.010.

Because T cell infiltration within the CRC TME has been closely linked to outcomes in patients, we first sought to establish whether there were sex-based differences in this parameter.^5,14,15^ We analysed T cells within multiple tissue compartments in tumour-bearing conditions to capture immune populations in tumoural, peritumoural, and systemic locations. To isolate the effects of sex on immune response, we used a syngeneic peritoneal metastasis model of CRC in age-matched male and female mice, of age-appropriate weight according to supplier.^16^ Mice were injected intraperitoneally with MC38 cells on Day 0 of the experiment. Tumours formed on the mesentery and peritoneal lining. Females survived longer by an average of 3.6 days, a 20.7% increase in mean survival time, and a 27.3% increase in median survival time compared to males (Fig. 1a). There was a statistically significant difference in the survival curves for male and female mice (log-rank test, *p* = 0.0047). At d20, 75% of females and 20% of males were alive. After sacrifice, dissociated tumours, peritoneal washings, and splenocytes were collected and analysed by FCA to look at intratumoural, peritumoural, and systemic immune profiles, respectively. The FCA gating strategy is shown in Supplementary Fig. 1; all cells were gated on size, singlet status, and viability before analysis for antibody binding. To confirm that the female survival was not due to decreased tumour proliferation, we performed Ki67 staining on slides from male and female tumours. We found no difference in tumour cell proliferation (Supplementary Fig. 2).

CD8+ T cells can inhibit tumour growth and progression in CRC, and strategies for increasing CD8+ T cell infiltration have slowed tumour growth and extended survival in animal models.^17^ There was no difference between the sexes in CD8+ T cell levels within the peritumoural (peritoneal) compartment. Male mice had a small but statistically significant increase in CD8+ cells in the systemic (splenic) immune compartment (*p* = 0.002). However, in the TME, females had a 1.69-fold increase in CD8+ T cells (Fig. 1b, *p* = 0.002).

To confirm the observed CD8+ cell increase in tumours, we performed immunohistochemistry (IHC) on tumours from male and female mice and examined non-necrotic tumour areas. There were increases in CD8+ cells in tumours from female mice (Fig. 1c). Furthermore, mice with CD8+ T cell percentages greater than the mean of the entire group survived an average of 21% longer than those with lower levels (Fig. 1d, *p* = 0.010).

### CD4+ T cell infiltration and differentiation differ between the sexes

The role of CD4+ T cells in CRC is complex, dependent on phenotype, and remains a subject of ongoing investigation. While higher total T cell counts within CRC correlate strongly with survival in human samples, CD4+ T cells have been shown to have both pro- and anti-tumour behaviour in CRC.^5^ The effects of CD4+ T cells on CRC are most commonly thought to be enacted through their capacity to alter both the adaptive and innate immune responses in the TME, as well as the production of cytokines that affect the entire TME response.^18^ Different CD4+ T cell phenotypes have been correlated with improved or worsened outcomes in this disease, with a clear picture of the most advantageous profile yet to emerge.

**Fig. 2:**
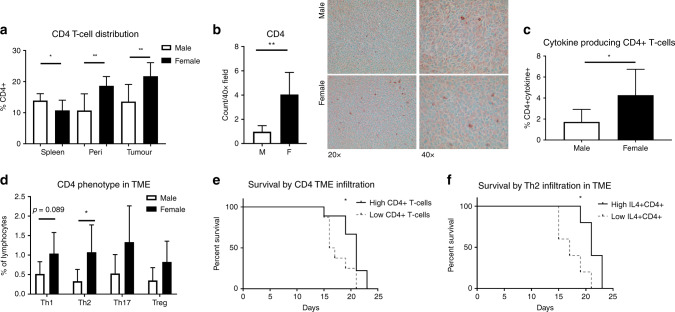
CD4+ T cell infiltration and cytokine production are increased in the female TME and correlate with survival. Cells from the spleen, peritoneal wash, and dissociated tumour were stained by flow cytometry. Cytokine expression characteristic of CD4+ T cell phenotypes was determined by CD4+ co-expression with characteristic cytokines (IFNy - Th1; IL-4 -Th2; IL-17A - Th17; IL-10-Treg). **a** CD4+ cells are increased in the peritoneal washes and tumours of female mice, as measured by FCA. **b** IHC using anti-CD4 shows more CD4 infiltration into female tumours. **c** Using FCA, females have a higher percentage of cytokine-producing CD4+ T cells than males in their TIL, with **d** elevated Th2 (IL-4+ CD4+) subpopulation. **e** CD4+ infiltration above the group mean correlated with longer survival, as did **f** increased proportions of Th2 cells. *p < 0.050; **p < 0.01; *n* = 8 for **a**, **e**; *n* = 5–9.

While CD8+ T cells varied within the TME and not the surrounding peritumoural environment, we found differences between the sexes in terms of their CD4+ T cell response to CRC in all compartments examined (Fig. 2a). Males had higher splenic populations of CD4+ T cells (*p* = 0.040), but lower populations in both the peritoneal cavity and the TME (*p* = 0.004 and 0.006, respectively). IHC confirmed elevated CD4+ cells in the non-necrotic areas of tumours from females (Fig. 2b). To see whether there was a difference in active, cytokine-producing cells, we examined the CD4+ T cells by FCA. To measure the production of cytokines characteristic to Th1, Th2, Th17 and Treg populations in the TME, cells were stained for intracellular IFNγ, IL-4, IL-17A and IL-10, respectively. Females had 2.47-fold higher active CD4+ T cells compared to males (Fig. 2c, *p* = 0.030).

When cytokine-producing CD4+ T cells were divided into major phenotypes, evidence of a greater local Th2 response was seen in the female mice. Females had increased percentages of IL-4 + CD4+ T cells in TME by 3.3-fold (Fig. 2d, *p* = 0.030). We also observed a trend toward increased levels of Th1 (IFNγ+ CD4+) T cells (*p* = 0.089).

We found that mice with higher (above average) TME CD4+ T cells survived 20.3 days, compared to 17.6 days in tumours with lower infiltration (Fig. 2e, *p* = 0.048). Furthermore, increased IL-4 + CD4+ TME infiltrate also correlated with increased survival (20.7 days vs. 17.8 days, Fig. 2f, *p* = 0.027). Peritoneal CD4+ T cell levels were closely correlated with TME CD4+ T cell levels and were associated with longer survival when elevated as well (21 days vs. 17.4 days, *p* = 0.031, data not shown).

We examined mRNA data from The Cancer Genome Atlas (TCGA) to see if our findings in this model were reflected in human data. Patient characteristics are in Table S1 (“Supplementary Material”). No differences in CD4 or CD8 mRNA expression were observed between men and women with CRC (data not shown). However, tumours showed sex-associated differences in both GATA3, a transcription factor for Th2, and Tbet, a transcription factor for Th1 and CD8 activation (Supplementary Fig. 3). GATA3 mRNA rose in women and Tbet mRNA decreased in men compared to normal tissue.

Taken together, these data indicate that the adaptive immune response to metastatic CRC is significantly different between the sexes, particularly within the TME. They support the hypothesis that the female adaptive immune response to metastatic CRC is more active than the male response. Furthermore, the correlation of the female pattern of adaptive immune response with increased survival suggests a potential mechanism for further investigation into the observed sex-associated survival difference seen in CRC.

### Serum cytokine levels are not reflective of TME cytokine production in tumour-bearing mice and the response in both compartments differs by sex

**Fig. 3:**
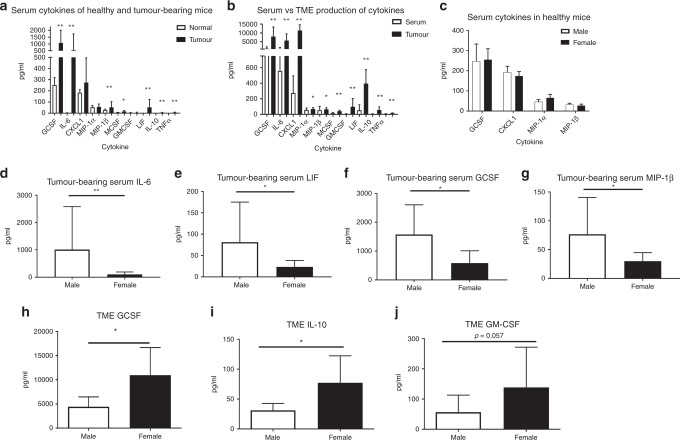
TME cytokine levels do not predict serum cytokine levels, and males and females have different serum and TME cytokine expression. Cytokines were measured via multi-plex bead-based array from healthy and MC38 tumour-bearing males and females, using serum or organ culture of tumour. **a** Tumour-bearing mice had elevated serum cytokine levels over healthy mice. **b** Serum and TME have different, non-correlated patterns of cytokine expression. **c** Sera from healthy mice has the same cytokine profile, but tumour-bearing males have elevated levels of **d** IL-6, **e** LIF, **f** GCSF and **g** MIP-1β. The TME of females had higher levels of **h** GCSF, **i** IL-10, and a trend toward **j** increased GM-CSF. (*p < 0.050, **p < 0.010). *n* = 5–9 between sexes and 12–18 between healthy and tumour groups.

Because cytokines and chemokines are both products of and shape the immune response, we characterised the levels both systemically and locally and then stratified by sex. To evaluate circulating systemic cytokine levels, we used serum; TME cytokine production was evaluated by culturing 8 mg pieces of tumour and analysing the conditioned culture medium by multiplex bead-based array. Based on observed changes in both the existing cell populations and cytokine production of T cells, we examined chemotactic and inflammation-associated cytokines. The addition of peritoneal CRC metastases resulted in significant shifts to the systemic profile. Healthy mice had low, sometimes undetectable levels of most cytokines, but increases were observed in tumour-bearing mice (Fig. 3a). For example, tumour-bearing mice had significant increases in the chemokines GCSF (*p* = 0.001), MIP-1β (*p* < 0.001) and MCSF (*p* = 0.048). Inflammation-associated cytokines increased in the sera as well, with significant differences in IL-6 (*p* < 0.001), LIF (*p* = 0.002), IL-10 (*p* < 0.001), and TNF-α (*p* < 0.001).

We compared systemic to local cytokines in tumour-bearing mice, anticipating that, generally, the serum would have lower concentrations of most cytokines compared to the TME (Fig. 3b). We did find decreased concentration of cytokines in the sera compared to the TME, including GCSF (*p* < 0.001), MIP-1α (*p* = 0.048), MIP-1β (*p* = 0.036), MCSF (*p* < 0.001), GM-CSF (*p* < 0.001), CXCL1 (*p* < 0.001), IL-6 (*p* < 0.001), LIF (*p* < 0.001), IL-10 (*p* < 0.001), and TNF-α (*p* = 0.003). However, the proportion of cytokine in tumour to sera varied from one cytokine to another; MIP-1α was only an average of 1.12-fold higher in the TME, while IL-6 was 10.29-fold higher. There was no significant correlation between systemic and TME levels of any elevated cytokines. This suggests that serum cytokine levels, while linked with prognosis in some cases, are not always directly reflective of the cytokine milieu of tumour cells.

Although healthy mice showed no difference in serum cytokines between males and females (Fig. 3c), tumour-bearing mice did. Specifically, sera from male tumour-bearing mice had higher levels of pro-inflammatory and chemotactic cytokines, including IL-6 (9.8-fold higher, Fig. 3d), LIF (3.5-fold higher, Fig. 3e), GCSF (2.7-fold, Fig. 3f), and MIP-1β (2.6-fold, Fig. 3g). In the TME, however, females had significantly elevated GCSF (Fig. 3h), and IL-10 (Fig. 3i), both by 2.47-fold. GMCSF levels were also increased in the female TME, although this did not reach statistical significance (2.45-fold, Fig. 3j, *p* = 0.057). These findings support the hypothesis that the systemic and local immune environment are different between the sexes in mice with CRC.

### Female mice have steeper cytokine gradients between periphery and TME

We observed that female mice had lower concentrations of serum cytokines than males, but simultaneously higher TME cytokines. This pattern was present in several cytokines, which led us to consider the steepness of the cytokine gradient between the systemic and the local environments in male and female mice. We compared female to male expression (e.g. female GCSF concentration for each sample/mean male GCSF concentration) in serum and tumour. Sera levels were used to look at circulating cytokines, and tumour was used to determine TME production of cytokines. By comparing the sexes and environment, we examined the potential gradient from the systemic to local cytokine/chemokine milieu. This gradient could contribute to the differences in the immune cell makeup of the TME between the sexes.

**Fig. 4:**
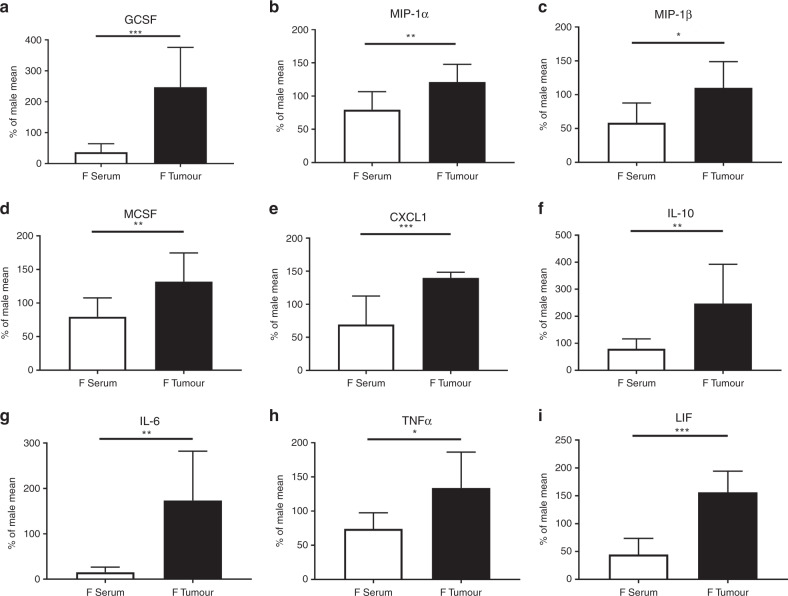
Female mice have steeper cytokine gradients between periphery and the TME. Females show significant difference in the relative quantity of cytokines in the serum and organ culture as compared to males. These values tend to be reduced compared to males in the serum but increased in the TME. The pattern is seen in chemotactic cytokines **a** GCSF, **b** MIP-1α, **c** MIP-1β, **d** MCSF and **e** CXCL1, as well as in **f** IL-10, **g** IL-6, **h** TNF-α and **i** LIF. Values are expressed as a percent of the cytokines from female compared to the male mean in the same location (*p < 0.050, **p < 0.010, ***p < 0.001). *n* = 7–9.

Cytokines that were relatively low in the periphery and high in the TME for females included macrophage chemoattractants. This cluster of chemokines included GCSF (*p* < 0.001, Fig. 4a), MIP-1α (*p* = 0.008, Fig. 4b), MIP-1β (*p* = 0.023, Fig. 4c), MCSF (*p* = 0.011, Fig. 4d), and CXCL1 (*p* < 0.001, Fig. 4e). The high tumour expression and low serum expression means that the cytokine gradient in females is steeper for many chemotactic cytokines, which could promote immune cell infiltration. Characteristic inflammation-associated cytokines also differed, with a similar low-systemic but high-TME pattern in the females. IL-10 (*p* = 0.005, Fig. 4f), IL-6 (*p* = 0.001, Fig. 4g), TNF-α (*p* = 0.012, Fig. 4h), and LIF (*p* < 0.001, Fig. 4i) had deviations from the male pattern of cytokine expression between circulating and local cytokine production.

These data suggest a stronger but more localised immune response to metastatic CRC in the females, accompanied by increased chemotactic cytokines, which could alter both the infiltration and behaviour of TIL and form the basis of a different immune microenvironment.

### Tumours from females trend toward increased IL-10 + macrophages, which correlated with increased survival

Because we observed differences in chemokines associated with macrophages in female mice, we investigated macrophage populations in the TME, peritoneal cavity, and spleen as well. Based on the cytokine findings, we expected a larger infiltrate of macrophages within the tumours of the female mice. Given the significant elevation of Th2 cells, IL-10, and GCSF within the female TME, we anticipated increased alternatively activated macrophages (AAM), which produce IL-10 and are associated with the Th2 axis. This hypothesis was partially confirmed.

**Fig. 5:**
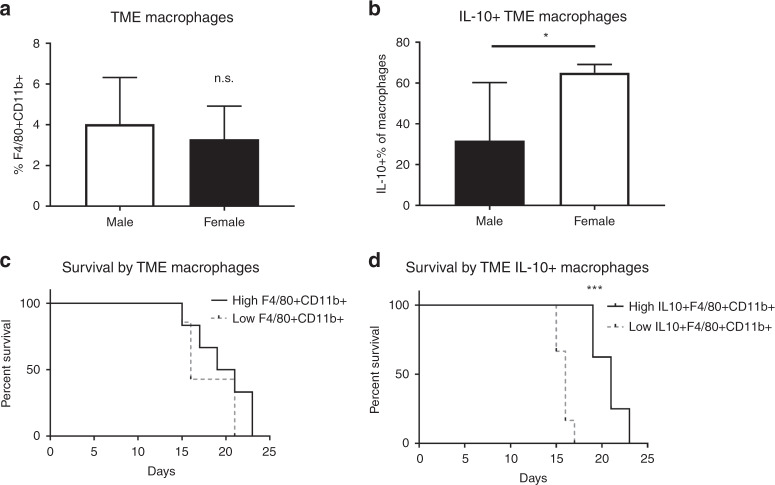
Survival correlates with IL-10-producing macrophages. FCA was performed on dissociated tumour, stained for macrophages that were F4/80 + CD11b+. **a** No difference was observed in total TME macrophage populations. **b** Females have a higher percentage of macrophages (F4/80 + CD11b+) that are producing IL-10 in the TME. **c** Mice with above mean macrophages in the TME had no survival benefit, but **d** mice with above mean IL-10 macrophages survived longer. *p < 0.050, **p < 0.010, ***p < 0.001; *n* = 6–8.

Male and female tumour-bearing mice had no significant difference in the proportions of macrophages within the TME (Fig. 5a). However, we did observe a tendency towards increased IL-10 producing macrophages (AAM) (IL10 + CD11b + F4/80+) in the TME (Fig. 5b). While total macrophage infiltration did not correlate with survival (*p* = 0.286, Fig. 5c), infiltration of AAM was strongly correlated with increased survival (*p* < 0.001, Fig. 5d). Animals above the group mean lived an average of 4.9 days, or 31.1% longer, than those with a percentage of AAM below the mean. These data indicate that the local macrophage immune response to metastatic CRC differs between the sexes in a survival-correlated manner.

## Discussion

The adaptive and innate immune response to metastatic CRC differs both systemically and locally depending on sex. Female tumour-bearing mice had increased CD8+ T cells within tumours which correlated with improved survival. In addition, tumours taken from female mice had significantly more IL-4 + CD4 + T cells, also correlated with increased survival. While healthy male and female mice had similar serum cytokines, the introduction of tumour led to significant differences between the sexes, with males having higher levels of pro-inflammatory cytokines. Examination of the secreted cytokines from tumour-bearing tissue, however, demonstrated a different pattern, with female mice having higher levels of chemotactic and inflammatory cytokines in this compartment, thereby creating a pattern of low systemic but high local immune response. The increased GCSF and IL-4 + CD4 + T cells in the TME, along with a trend toward increased AAM, is evidence of a local Th2-oriented response to CRC in the females. This pattern also correlated positively with survival. These data reveal a role for the local immune response in females in contributing to the prolonged survival that we observe in our model and in human patients. As noted, some of the aspects of the female immune response, such as the increased T cell infiltration, have been strongly correlated with improved survival in patients;^5^ unfortunately, the referenced study only adjusted for sex as a confounding factor, and did not formally compare Immunoscores between males and females in CRC. Our findings suggest that T cell-related markers should be evaluated for sex-associated differences.

CD8+ infiltration and activation are important prognostic markers for CRC survival, which aligns with the observation that the female mice had increased, survival-correlated CD8+ T cells in the TME.^19^ Tumour-specific CD8+ T cells can drive anti-tumour responses and are understood to be vital for immune checkpoint inhibition therapies.^19–21^ In CRC, most CD8+ T cells are exhausted in tumours, potentially making CRC a strong candidate for immune checkpoint therapy.^22^ Response to checkpoint therapies have been promising in microsatellite instable CRC, which is correlated with increased infiltration of CD8+ T cells.^23,24^

However, our study suggests that the existing T cell environment may differ by sex. If the female TME is able to extend the period of anti-tumour CD8+ activity, perhaps through support from the observed increase in Th2 response, that could be one reason for their increased survival. It could also signal that a sex-stratified response is possible in CRC immunotherapy. However, different tumour types and metastatic niches may hold unique immune responses; further investigation into additional models, as well as primary TME, is warranted. These findings underscore the importance of including both sexes in study designs.

The clinical relevance of these findings is supported by some data from TCGA. Although we did not see differences in CD4 and CD8 mRNA in TCGA CRC data, these proteins have been found to not reliably correlate with mRNA levels.^25^ However, markers of T cell activity did shift in a way consistent with the animal model. The finding of elevated GATA3 in women and decreased Tbet in men (compared to normal tissues) is intriguing, and would be consistent with a stronger Th2, Th1 and/or CD8 anti-tumour response in women. If the differences noted in our model are confirmed in human data, women may be more responsive to therapies that increase T cell responses, particularly CD8+ T cell responses.^22,26^ Anti-PD-1 has been shown to be more effective in melanoma in patients with high CD8+ cell infiltration, the phenotype we have observed in the female TME.^27^ On the other hand, these differences may be highly sensitive to tumour origin and metastasis location. Early results suggest men are responding better to immune checkpoint blockade therapy than women in general, but these benefits vary by tumour type.^28–30^ This suggests that sex may affect the immune response in other cancers as well, potentially in a tumour-specific manner that requires close examination.

Depending on phenotype, CD4+ T cells can drive or suppress tumour responses. We found total TME CD4+ T cells to correlate with survival, and there was significantly more active CD4+ T cell infiltration in female TME. CD4+ T cells can shift the cytokine milieu significantly, depending on phenotype, potentially explaining in part the differences between the sexes in cytokine levels within the TME. The survival benefit observed for increased total TME CD4+ T levels was correlated with Th2, but not Th1, Th17, or Treg accumulation. This is consistent with our finding of increased GCSF in tumours from female mice, as GCSF supports increased Th2 polarisation.^31^ While IL-4, produced by Th2 cells, has pro-tumour behaviour, particularly by supporting cancer stem cells,^32^ tumour-specific Th2 responses can also help eliminate tumours by reinforcing anti-tumour CD8+ T cell activity.^33^ We found that the increased Th2 and Th2-associated AAM correlated with improved survival, supporting the hypothesis that the Th2 axis within metastatic CRC could support anti-tumour immune activity.

In much of the literature Th1 cells have been demonstrated to have anti-tumour responses, particularly because they can support CD8+ cytotoxicity. However, Th2 cells are less resistant to anergy than Th1 cells, and their anergy is more reversible.^34^ This anergic resistance may be crucial in long-term anti-tumour T cell responses by extending the lifetime of beneficial T cell activity, particularly as T cell anergy can blunt anti-tumour immune responses.^22^ The increase in survival observed in conjunction with increased Th2 infiltration suggests that their activity may play an important role in anti-tumour responses in metastatic CRC. One potential arena that Th2 cells may be supportive in is CD8 cytotoxicity. Like Th1 cells, Th2 cells can be synergistic in assisting anti-tumour CD8+ T cell responses, as shown by a study that examined co-infiltration of CD4+ phenotypes with CD8+ T cells in a model of brain cancer.^35^

The sex-based differences between local and systemic cytokine levels seen in this model support consideration of the cytokine and chemokine gradients that may affect immune cell infiltration or activation. Cellular responses to signalling, including chemotaxis, are affected both by concentration and the steepness of cytokine gradients.^36^ In this model, we identified cytokines that were high in the TME and low in the sera for females with metastatic CRC, as compared to males. These cytokines promote activation or chemotaxis, particularly of macrophages. GCSF, elevated in male sera and female TME, supports macrophages that express M2-like markers, including IL-10 production.^37^ A subset of AAM macrophages, M2a, increases Th2 differentiation. We saw increased IL-10 in the female TME, which increases the activity of Th2-differentiating macrophages as well as directly increasing Th2 activity.^38^ These findings parallel observed behaviour in sepsis, where there is an increase of macrophages and macrophage activity locally, but a lower systemic inflammatory response in females.^7^ The importance of various macrophage phenotypes is not fully understood in CRC, and further study into the activity of these cells will increase our understanding of the basis of their potential benefit and role in the TME. Indeed, the AAM may be contributing to the increased Th2 activity we observed in the TME of females in this model.

These findings support an immunological basis for the sex-based differences seen in CRC. Others have reported that sex interacts with other aspects of CRC presentation and progression. For example, metabolic status seems to exacerbate existing differences in tumour site between men and women,^39^ and it is well established that premenopausal females possess greater metabolic resiliency as compared to age-matched males.^40–42^ Some studies report an advantage for younger women in CRC, suggesting a hormonal mechanism, but postmenopausal women also have improved overall survival.^3^ Hormone replacement therapy reduces the risk of developing CRC and reduces mortality at both early and late stage cancer.^43,44^ Improving understanding of how the immune response is affected by multiple overlapping systems, including the tumour itself, remains an ongoing research challenge.

One unanticipated finding was the difference in cytokine patterns between serum and the TME. Because of the difficulty of accessing and sampling the TME, studies have used serum cytokines as indicators for what may be happening in the tumour. However, we show that a cytokine can be elevated in the serum, but depressed within the tumour, and vice versa; in fact, this pattern is a characteristic difference between male and female immune responses to tumours in our model. It remains to be seen if this pattern holds true in human patients; however, using systemic cytokine levels as an indicator of the inflammatory signalling most relevant to the tumours could be misleading. That does not necessarily imply that serum cytokines cannot be used as a prognostic tool, but rather that conclusions regarding the TME based only on examination of the sera are likely to be incomplete, particularly for females.

Our findings demonstrate fundamental differences between the TME of females and males with metastatic CRC. The tight correlation of survival with changes in various immune compartments we observed suggest that the differences we have found are relevant to outcomes. While the mechanisms behind the different immune responses require more study, these data support the need for including sex as a variable in immunologic studies of CRC. Moreover, experimental designs that include intact and gonadectomised animals may also provide insight into sex differences in CRC outcomes. Because the immune system alters responses to therapies such as immune checkpoint blockade, results from men and women may vary because of pre-existing differences in the TME. These data may also be of relevance for those looking for the source of the survival benefit that women have for this disease. With further study, manipulation of the immune environment of both men and women may be able to improve outcomes even further.

## Supplementary information

Supplementary Material

## Data Availability

The datasets used in the current study are available from the corresponding author on reasonable request.
