# Supplementary material for: The role of sex in the innate and adaptive immune environment of metastatic colorectal cancer
Source: Br J Cancer. 2020 May 26;123(4):624–32. doi: 10.1038/s41416-020-0913-8 (PMC7435180; doi:10.1038/s41416-020-0913-8)
Supplement: Supplementary file 1 — Supplementary Material [file 41416_2020_913_MOESM1_ESM.pdf]

Supplemental Figure Legends

Supplementary Figure 1. FCA gating strategy to identify viable T cells. A) All samples were singlet-gated by forward scatter for roundness to exclude debris. Then cells B) were gated on size. Splenocyte populations were used to create gates for expected sizes of lymphocytes, with the same gate shared between spleen, peritoneal wash, and dissociated tumor cells (from left to right). There was low prevalence (2-5%) of cells with immune markers outside these gates and marker positive cells were frequently positive for multiple contradictory markers (i.e. CD4+CD8+) or were gated under live/dead discrimination and considered likely autofluorescent. C) Cells were gated on viability using TonboBio GhostDye. D) Representative CD4 vs CD8 plots of cells gated for size and viability from splenocytes, peritoneal wash, and dissociated tumor from male and female tumor-bearing mice are shown. E) CD8+ and CD4+ cells were verified to be CD3+.

Supplementary Figure 2. Ki67+ cells within tumors do not differ with sex. Tumors stained for Ki67 by IHC were then quantified using Celleste software for positive populations in non-necrotic portions of tumor tissue. A) Representative 20x picture of portions of tumor from female and male mice. B) Image analysis by Celleste counted Ki67+ populations and showed no difference in Ki67 staining between tumors from males and females. n=9-13.

Supplementary Figure 3. Sex-associated differences in T cell transcription factors. Colon adenocarinoma and normal tissue data were acquired from TCGA, and stratified by sex. A) GATA3 mRNA was increased in tumor tissues from women, while B) Tbet mRNA decreased in tumor tissues from men, compared to normal tissue from the same sex. * = $p < 0.050$, n=124-184

Supplemental Methods

Animal Husbandry

Mice were obtained directly from The Jackson Laboratory. Mice were housed in specific-pathogen free facilities, that met guidelines recommended by the U.S. National Research Council for the care of research animals, including climate control for temperature and humidity and an automated day/night cycle. Cages contained 2-5 littermates of the same sex, nesting material, 1-2 enrichment items, and free access to food and water. Corncob bedding was used.

Mice were kept in cages with access to enrichment and a least one same-sex littermate in climate-controlled SPF facilities. The mean weight at the beginning of the study was 16.2g for females and 21.3g for males, ±<5%.

Animals were euthanized with CO_2, per AVMA guidelines to minimize distress.

Ki67 Staining

Intraperitoneal tumors were harvested from male and female C57BL/6J mice, processed for immunohistochemistry, and stained with rabbit anti-Ki67 (ab16667, Abcam, Cambridge, MA, USA).

Sections for Ki67 were processed for immunohistochemistry using the ImmPRESS™ VR Horse Anti Rabbit IgG HRP Polymer kit (cat# MP-6401, Vector Labs, Inc., Burlingame, CA). Antigen retrieval (pH 6 Citrate

Antigen Unmasking Solution, cat# H3300, Vector Labs, Burlingame, CA) was accomplished via twenty minutes in a steamer followed by thirty minutes cooling at room temperature. Sections were treated with a peroxidase blocking reagent (Bloxall, cat# SP-6000, Vector Laboratories, Inc, Burlingame, CA) to inhibit endogenous peroxidase activity, followed by 2.5% normal horse serum to block nonspecific staining. Rabbit anti Ki67 (abcam, cat#16667, 1:200 dilution, Cambridge, MA) was applied to each section and following incubation overnight at 4°C in a humidified chamber, sections were washed in TBS and the ImmPRESS Polymer reagent was applied according to the manufacturer's direction.

Slides were incubated with NovaRed® (Vector Laboratories, Inc., Burlingame, CA) chromogen for visualization. Counterstaining was carried out with Methyl Green (Vector laboratories, Burlingame, CA). Appropriate positive and negative tissue controls were used.

Samples were imaged on an EVOS M7000 in the transmitted light channel using an EVOS 10x objective (Thermo Fisher Scientific, Waltham, MA, USA) and an Olympus 20x objective (Olympus, Tokyo, Japan). Quantitative analysis on the acquired tissue images was performed using the Smart Segmentation and Count features of Celleste Image Analysis software, version 4.1.1 (Thermo Fisher Scientific, Waltham, MA, USA). Cell nuclei in non-necrotic areas that were positively and uniformly stained were quantified as either Ki67 positive or negative based on stain intensity features using Smart Segmentation. The morphological watershed algorithm in the Count feature was used

to segment any clustered nuclei. Representative images were color-corrected slightly to adjust for camera color bias.

mRNA Datasets, Visualization and Statistical Analyses

The RNA-seq datasets and protein expression data for 131 proteins for colon adenocarcinoma (COAD) were downloaded from the University of California, Santa Cruz (UCSC) Cancer Genome Browser (xenabrowser.net). Only publicly available, deidentified data were accessed from TCGA for the analyses reported here. Basic characteristics of the patients used in the survival analyses are provided in Supplementary Table S1. The RNA-seq datasets for colon normal tissues (184 males vs. 124 females) from Genotyping-Tissue Expression (GTEx) were also downloaded from USCS browser. Basic statistical analyses and visualization were performed using R v3.6.1. Kaplan–Meier for patient survival were performed and visualized using the survival and survminer packages in R. For nonparametric comparisons, the Wilcox test was used, $p < 0.05$ was considered to be statistically significant.

Table S1. Clinical patient characteristics for the colon cancer

<table>
<tr><th>Characteristics</th><th>N</th><th>OS</th></tr>
<tr><td>Age (year)</td><td>65</td><td>HR = 0.69(0.42|1.1) P = 0.154</td></tr>
<tr><td>>65</td><td>161</td><td></td></tr>
<tr><td><65</td><td>125</td><td></td></tr>
<tr><td>Gender</td><td></td><td>HR = 1.4(0.88|2.3) P = 0.152</td></tr>
<tr><td>Male</td><td>154</td><td></td></tr>
<tr><td>Female</td><td>134</td><td></td></tr>
<tr><td>Disease Stage*</td><td></td><td></td></tr>
<tr><td>Stage I</td><td>44</td><td></td></tr>
<tr><td>Stage II (vs. Stage I)</td><td>110</td><td>HR = 2.1 (0.63|7.2) P = 0.22</td></tr>
<tr><td>Stage III (vs. Stage I)</td><td>82</td><td>HR = 3.7 (1.12|12.5) P = 0.032*</td></tr>
<tr><td>Stage IV (vs. Stage I)</td><td>40</td><td>HR = 9.3(2.76|31.6) P < 0.0001*</td></tr>
<tr><td>Stage T*</td><td></td><td></td></tr>
<tr><td>Stage I+II</td><td>50</td><td></td></tr>
<tr><td>Stage III (vs. Stage I+II)</td><td>196</td><td>HR = 1.9 (0.77|4.9) P = 0.162</td></tr>
<tr><td>Stage IV (vs. Stage I+II)</td><td>40</td><td>HR = 7.6 (2.77|20.6) P < 0.001*</td></tr>
<tr><td>Nodal Invasion*</td><td></td><td></td></tr>
<tr><td>N0</td><td>166</td><td></td></tr>
<tr><td>N1 (vs. N0)</td><td>71</td><td>HR = 2.1 (1.2|3.7) P = 0.01*</td></tr>
<tr><td>N2 (vs. N0)</td><td>49</td><td>HR = 3.4 (1.7|6.0) P < 0.001*</td></tr>
<tr><td>Distant Metastases*</td><td></td><td></td></tr>
<tr><td>M0</td><td>193</td><td></td></tr>
<tr><td>M1 (vs.M0)</td><td>40</td><td>HR = 4.3(2.4|7.5) P < 0.001*</td></tr>
<tr><td>MX</td><td>49</td><td></td></tr>
<tr><td>Tumor Site</td><td></td><td></td></tr>
<tr><td>Left</td><td>104</td><td>HR = 1.4(0.82|2.3) P = 0.221</td></tr>
<tr><td>Right</td><td>181</td><td></td></tr>
</table>

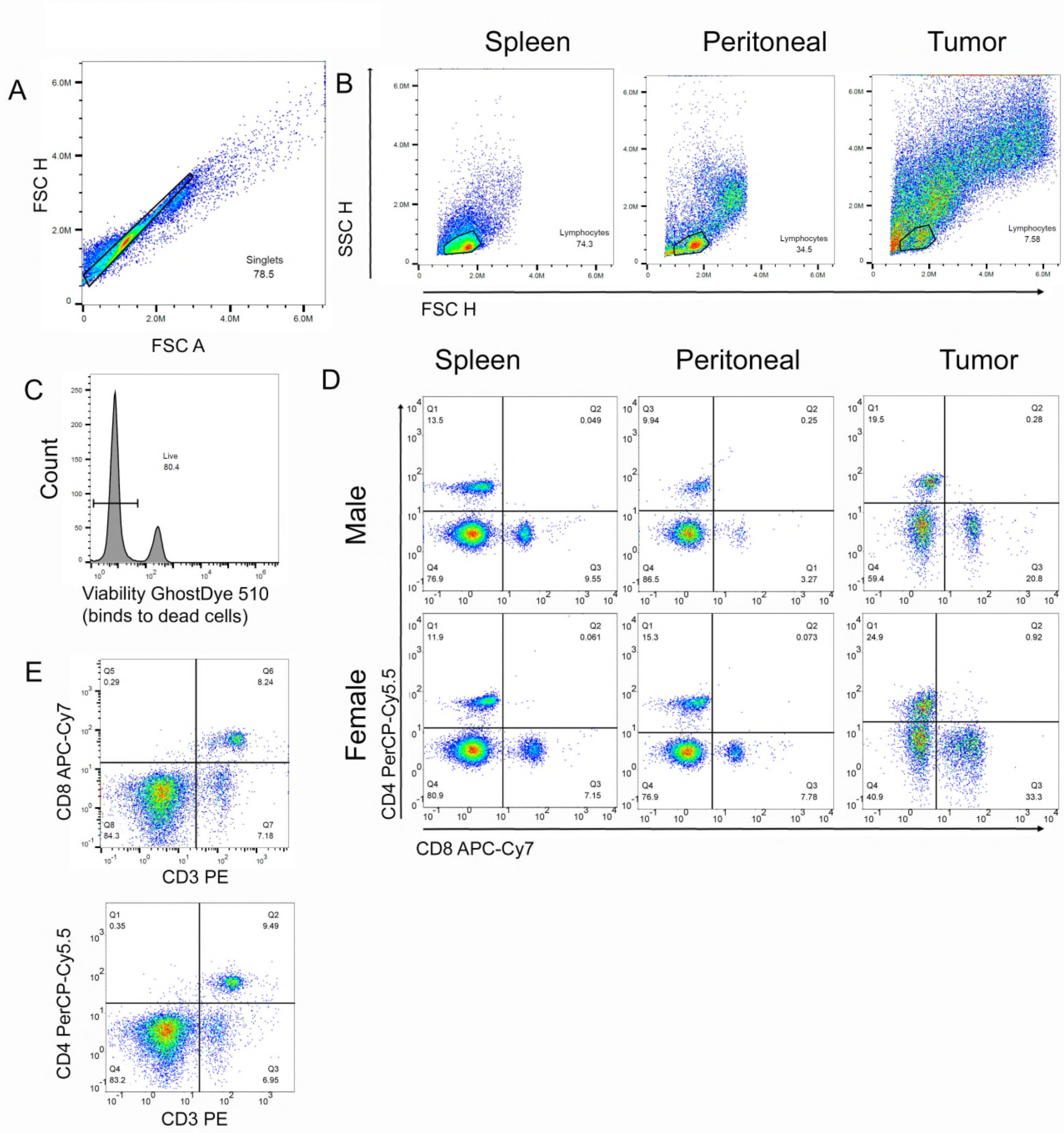

Supplemental Figure 1

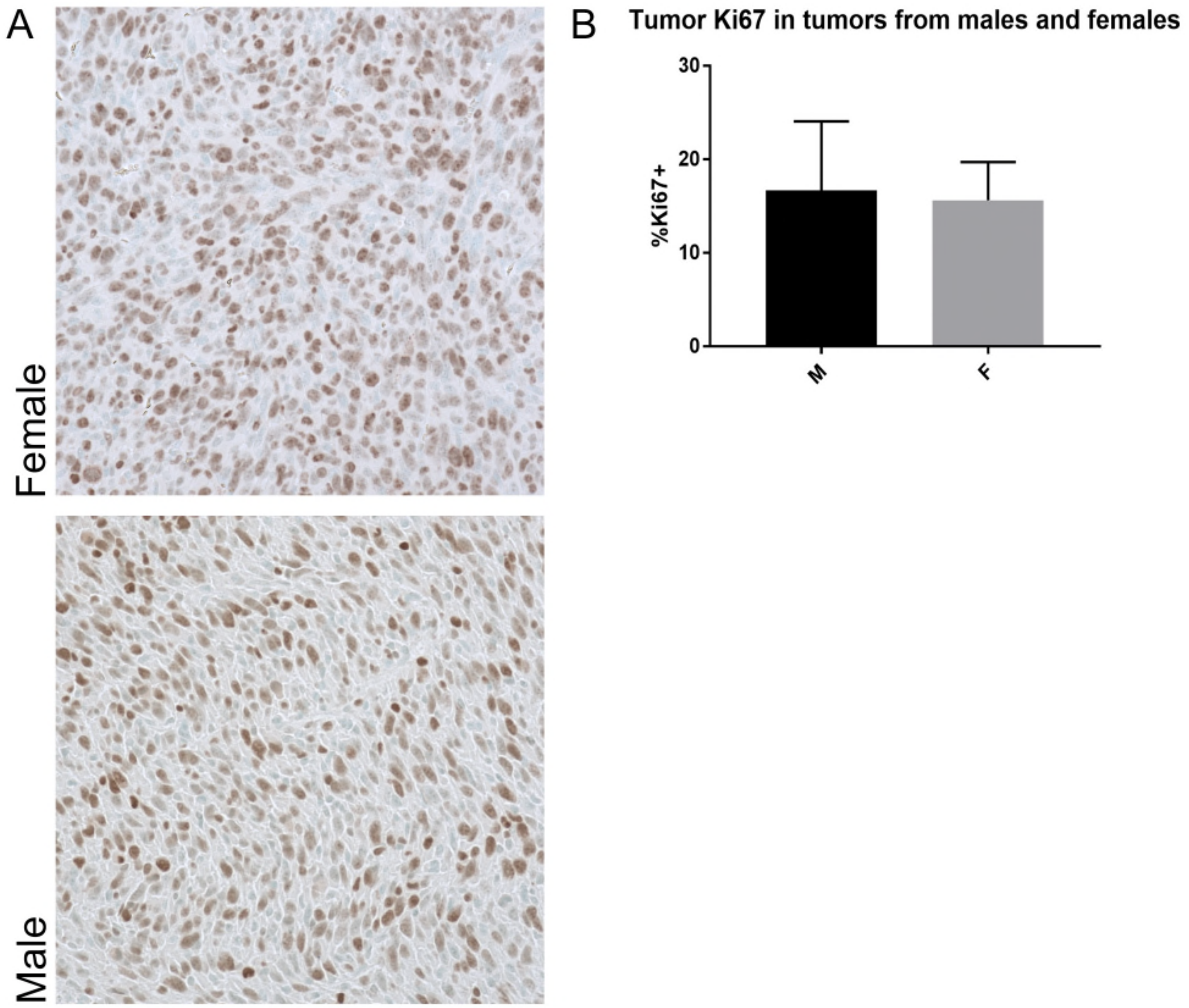

Supplemental Figure 2

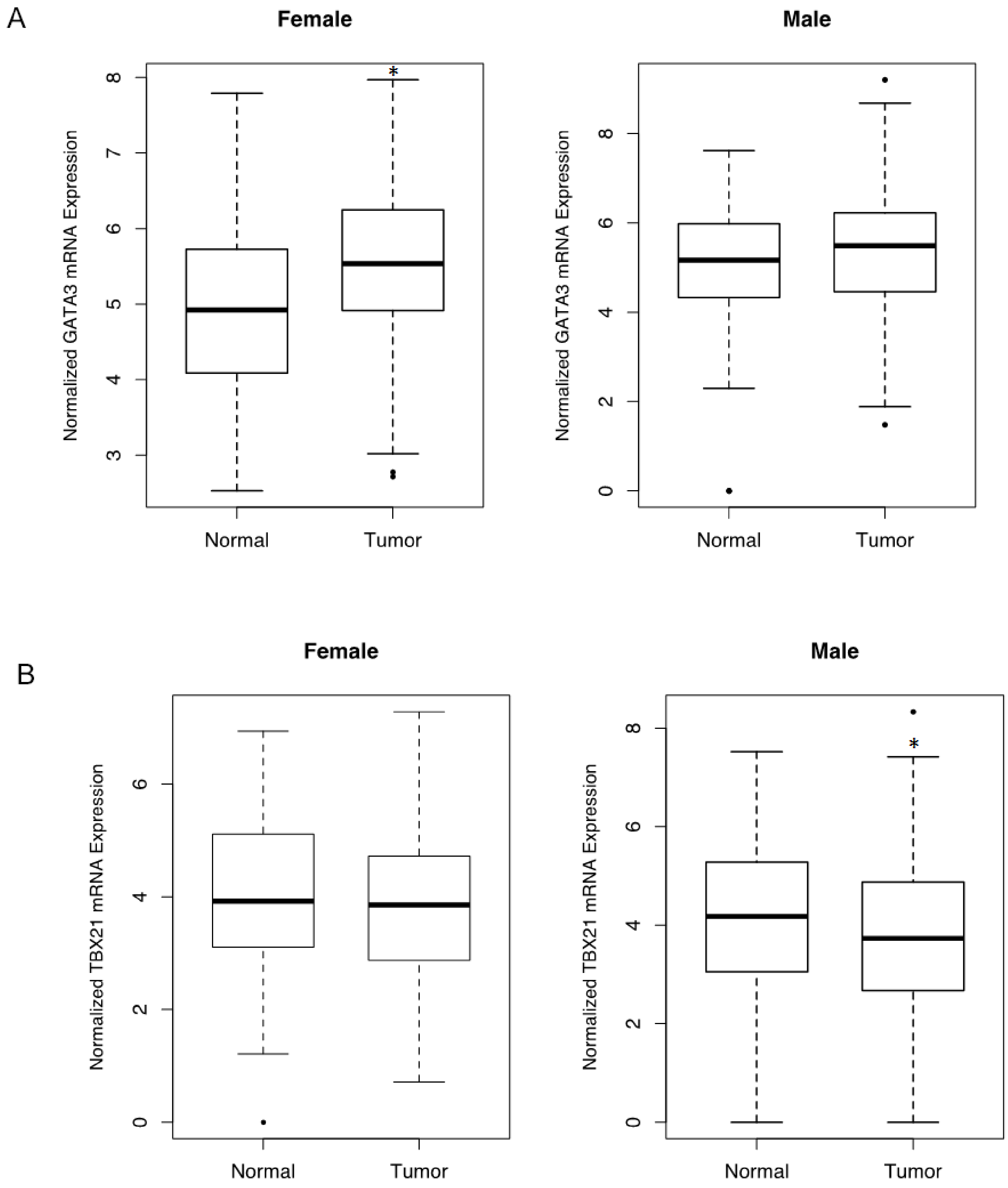

Supplemental Figure 3
